# Utilising a Real-Time Continuous Glucose Monitor as Part of a Low Glycaemic Index and Load Diet and Determining Its Effect on Improving Dietary Intake, Body Composition and Metabolic Parameters of Overweight and Obese Young Adults: A Randomised Controlled Trial

**DOI:** 10.3390/foods11121754

**Published:** 2022-06-15

**Authors:** Khadidja Chekima, Mohd Ismail Noor, Yasmin Beng Houi Ooi, See Wan Yan, Mohammad Jaweed, Brahim Chekima

**Affiliations:** 1 Faculty of Health and Medical Sciences, Taylor’s University, Selangor 47500, Malaysia; khadijachekima@gmail.com (K.C.); yanseewan@gmail.com (S.W.Y.); mohammad.jaweed@taylors.edu.my (M.J.); 2 Faculty of Medicine and Health Sciences, The National University of Malaysia, Selangor 43600, Malaysia; ismailnoor49@gmail.com; 3 Faculty of Food Science and Nutrition, University Malaysia Sabah, Sabah 88450, Malaysia; yasmin@ums.edu.my; 4 Faculty of Business, Economics and Accountancy, University Malaysia Sabah, Sabah 88450, Malaysia

**Keywords:** real-time continuous glucose monitoring (rtCGM), low glycaemic index, low glycaemic load, weight loss, obesity management, overweight and obesity, young adults

## Abstract

A randomised controlled trial to measure the effects of integrating real-time continuous glucose monitor (rtCGM) into a low glycaemic index (GI) and glycaemic load (GL) dietary intervention on dietary intake, body composition and specific metabolic parameters was carried out. A total of 40 overweight young adults [(means ± SD) age: 26.4 ± 5.3 years, BMI: 29.4 ± 4.7 kg/m^2^] were randomised into an intervention and control groups for a period of eight weeks. Both groups received nutrition education on low GI and GL foods. The intervention group also received an rtCGM system to monitor their glucose levels 24 h a day. While controlling for physical activities and GI and GL nutrition knowledge, the results indicated that the rtCGM system further improved body weight, BMI, fat mass, fasting plasma glucose, HbA1c, total cholesterol, HDL cholesterol and LDL cholesterol in the intervention group (*p* < 0.05). This trial unveils the robustness of the rtCGM where non-diabetic overweight and obese young adults can benefit from this device and utilise it as a management tool for overweight and obesity and a primary prevention tool for type 2 diabetes, as it provides real-time and personalised information on physiological changes.

## 1. Introduction

Optimising individuals’ adherence to a recommended nutrition intervention has been a continuous challenge faced by researchers, dieticians and nutritionists. Despite the fact that lifestyle changes programmes are highly effective, their efficacy is frequently reinforced by intensive methods requiring regular monitoring and support from health experts to ensure adherence and intended outcomes. These resource intensive approaches limit their applicability to a broad range of situations where the number of allied health professionals are not meeting the minimum required number suggested. According to the World Health Organisation, the threshold of health professionals for every 100,000 population is 2.3 [1]. However, many countries, especially in Africa and Asia, do not meet the minimum number of health professionals.

In order to enhance the efficiency of diet-related intervention to achieve the desired outcomes for a specific target population, there needs to be more effective strategies which involve the development of a more real time, personalised dietary intervention which could be achieved using validated digital health tools [2]. It has been proven that self-monitoring of health markers and behaviour outside of a clinical setting has been shown to be an effective approach for evaluating treatment response and enhancing adherence for a number of health outcomes, including body weight [3]. Real-time continuous glucose monitor (rtCGM), a type of digital health tool classified under decision support systems, has been approved by the FDA, which ensures its safety for use. Studies have shown that this device has helped in managing diabetes by improving glycaemic control, reducing hypoglycaemic events, reducing body weight and calorie intake, increasing adherence to eating plans and several other health improvements [4].

With the existing evidence, it is most relevant to determine the benefits of this device among individuals who are at risk of developing diabetes. In this study, we aimed to modify the dietary intake of overweight and obese young adults into practicing a dietary intake of low GI and GL foods driven by the use of rtCGM as a motivating tool that provides the individuals real time, personalised information of their daily glucose levels.

## 2. Materials and Methods

Ethics approval was obtained from Taylor’s University Human Ethics Committee (TUHEC) of Taylor’s University. The study was conducted in accordance with guidelines of the Helsinki Declaration and was registered with the UMIN Clinical Trials Registry as UMIN000047556. Individuals who volunteer to be participants of this study were given a Subject Information Sheet which contains the descriptions of the studies. Individuals were allowed to ask questions. The individuals were also verbally informed with full details of the studies protocols. Once the individuals agree to participate in the studies, they were asked to sign the consent form.

### 2.1. Study Design

**Figure 1:**
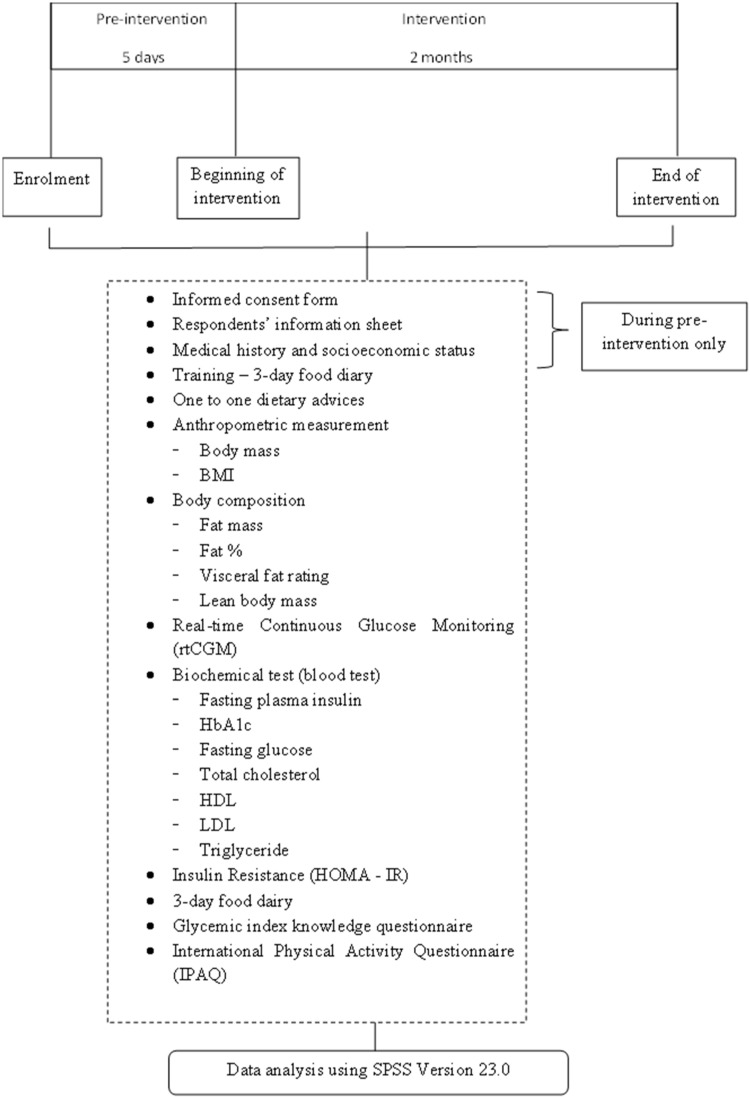
Study protocol.

The design of this study is an RCT which involves two phases, namely the pre-intervention phase and the intervention phase, as shown in the study protocol in Figure 1. Participants were randomly assigned using random.org/sequences/ (accessed on 18 October 2021) to either the intervention group or the control group. Both groups received nutrition education aimed at modifying daily dietary intake to low GI and GL foods. For the intervention group, the rtCGM system was used, where participants received an rtCGM sensor and an rtCGM reader to observe their glucose levels 24 h a day. Each participant received two sensors, each with a lifetime of 14 days. The sensors were worn during the first and last 14 days of the intervention. The control group, however, did not use an rtCGM system, and thus, participants were not able to observe their glucose changes. The use of the rtCGM system enabled the intervention group to receive real time, on demand and personalised information on their current glucose level, its direction and rate of change, which may prompt them to make positive food dietary changes. At both phases, specific parameters of the participants were measured. These include anthropometric measurement, body composition and specific metabolic parameters, which are listed in Figure 1. These parameters were compared before and after the intervention within and between groups. Detailed description of the intervention process flow and steps involved in both phases are described in the following sections.

### 2.2. Phases of Intervention

#### 2.2.1. Screening of Participants

**Figure 2:**
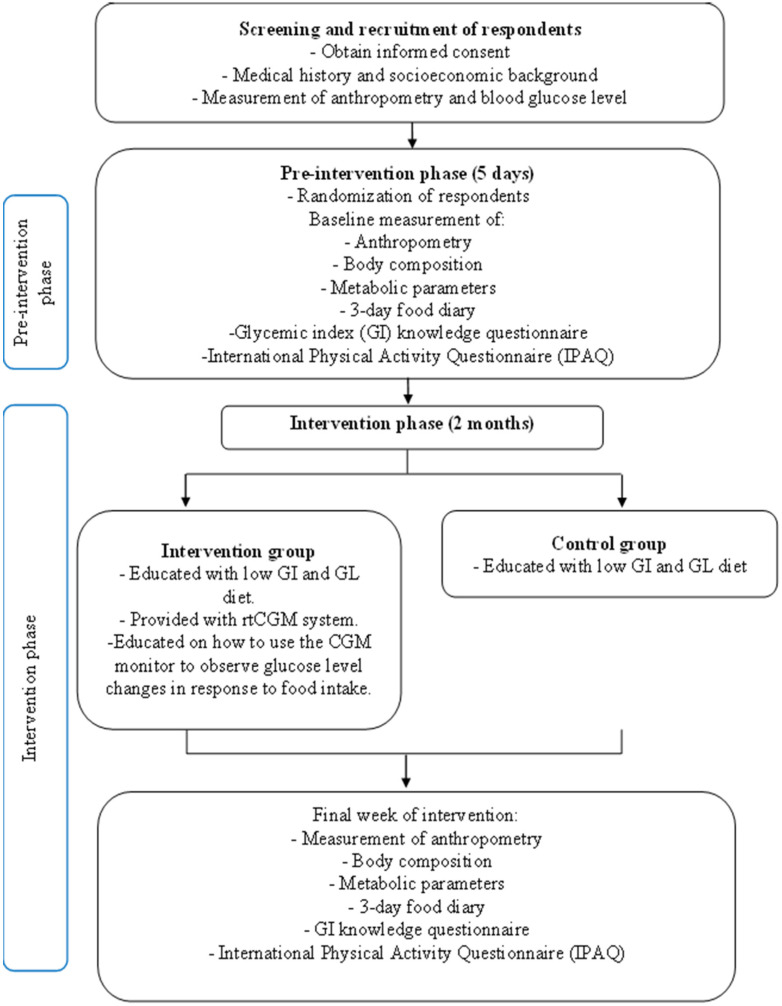
Intervention flow diagram.

The phases of the intervention are summarised in Figure 2. Prior to recruitment, potential individuals were required to provide their consent to be part of the study by signing the consent form. They were then screened according to the inclusion and exclusion criteria as elaborated. The inclusion criteria were overweight or obese individuals between the age of 18 to 35 years old, non-smoking (defined as ≤1 cigarette/day), consuming ≤14 alcoholic drinks/week (1 drink is equivalent to 10 g alcohol), stable body weight (±3 kg) for the past 3 months and normal fasting blood glucose (<5.6 mmol/L). The exclusion criteria were lactating or pregnant women, following a vigorous weight loss regimen, suffering from diabetes, hepatic or renal dysfunction, having had a bariatric surgery, taking medication or supplementation that affects appetite or food intake and using medication which may affect glucose metabolism (e.g., corticoids, thyroid hormones and thiazide diuretics) [5]. Details on medical history was obtained and body composition and blood glucose level were measured. Individuals who passed the screening criteria were recruited as the participants of this study and were randomised to be part of the intervention group or the control group.

#### 2.2.2. Pre-Intervention Phase

At this point, the pre-intervention phase of a duration of five days begun. Participants were given training on food dietary record as they were instructed to complete a 3-day food diary at specific times during the intervention period. During the pre-intervention phase, a 3-day food diary was reported from day two to day four. Anthropometric measurements, body composition, specific metabolic parameters, glycaemic index knowledge and physical activities data were collected from the participants as baseline data. At this point, participants from the intervention group began wearing the rtCGM. At the pre-intervention phase, the participants were blinded to the rtCGM data. They were required to scan the sensor at least four times a day but were not taught the meaning of the glucose levels and the screens of the rtCGM readers displaying the glucose levels were covered to ensure blinding of participants.

#### 2.2.3. Intervention Phase

The intervention then took place for a duration of two months. Based on a review on low GI and GL diet intervention studies among overweight and obese individuals, a duration of four weeks was sufficient to observe significant body weight changes [6]. The randomised participants to the intervention and control groups were given nutrition education related to low GI and GL diet. Participants from the intervention group continued wearing the rtCGM sensors to record their glucose levels during the intervention period. However, they were no longer blinded to the rtCGM data and were allowed to observe their real-time glucose reading at any point of the day.

They were guided on how to use the rtCGM reader and how to utilise the device to make more informed decisions on choosing lower GI and GL foods. The intervention group were able to observe the significant fluctuation of glucose level upon consumption of higher GI or GL foods as compared to a steadier increase of glucose level when consuming lower GI or GL foods. The real-time feedback of the rtCGM reader allowed them to be more conscious of the food choices they make as an individual’s glucose changes can be instantly observed through the rtCGM.

Anthropometry, body composition and metabolic parameters were collected on day 45, whereas a 3-day food diary was kept from day 43 to day 45. Their GI knowledge and physical activities were re-evaluated once again at the end of the intervention.

### 2.3. Sampling

The sample size was estimated using G* Power software (University Düsseldorf, Düsseldorf, Germany, version 3.1) [7]. The sampling size was determined based on changes in the primary outcome which is changes in body weight. A power of 90% with a significant difference (*p* = 0.05) to detect a difference of two kg in body weight change among groups with a standard deviation of two was used to calculate the sample size [8]. Based on the calculation, it was estimated that 18 participants were required for each group (intervention and control group). Accounting for a 10% dropout rate, the total number of participants required for each group is 20. Thus, a total of 40 participants were recruited for this study.

### 2.4. Dietary Intervention

In this study, a low glycaemic index and load nutrition intervention for overweight and obesity was designed for the participants. The intervention included the definition of obesity as a disease and low GI and GL, the risk factors associated with obesity, the severity of this disease, the benefits of practicing low GI and GL diet, ways to overcome the barriers to adapt a low GI and GL diet as a daily dietary habit, ways to include low GI and GL foods in the diet and approaches to improve self-efficacy.

A low GI and GL nutrition education focused on teaching participants to swap high GI and GL food to foods with lower GI and GL. This swapping method has been reported to be successful for interventions conducted in the Asian region [9,10,11] and other regions [12]. Information leaflet that contains a list of higher GI and GL foods versus their lower GI and GL counterpart was provided for the participants. This food list was divided into food groups, namely breads, oat, potatoes, rice, noodles, snacks and beverages.

The swapping of foods allowed greater flexibility for participants to choose from a variety of foods according to their preference, which may lead to a better adherence to the diet. To avoid burdening the participants, they were not required to memorise the numerical GI values of foods but were rather advised to include lower GI foods in every meal to substitute the common high GI food, such as white rice and bread, instant noodles and biscuits, with lower GI foods, such as brown rice, multigrain bread, wheat spaghetti, dhal and nuts. No explicit instructions for energy (kilocalories) restriction were given [13], but rather, this study relied on participants’ intrinsic control of energy intake based on the presumption that these diets would decrease hunger and increase satiation and/or satiety, and therefore, promote a negative energy balance [14].

Participants were also instructed to balance the consumption of macronutrient at every meal to achieve a lower GL diet [15]. They were led by three simple steps to follow when selecting or preparing a meal. The first step was to select lower GI carbohydrate, such as low GI rice, pasta, dhal or barley, and to avoid high GI foods, such as white rice, white bread or corn flakes. Secondly was to add some protein for fullness, such as marinated tofu, stir fry beef or chicken, steamed seafood or fish, tandoori chicken or sashimi. The third and final step was to fill at least half of the plate with vegetables.

In order to reduce the post-prandial blood glucose spikes upon consumption of high GI foods, participants were advised to combine high GI foods with vinegar, dairy products, pickles, proteins or fats. A number of scientific research papers have reported a reduction of the post-prandial glycaemic response upon consumption of higher GI foods in combination with these foods and macronutrients [15,16].

Nutrition education was provided one to one to the study participants during baseline and printed materials were used to illustrate the message and to serve as a reminder at home. A softcopy of the materials was sent to the smartphones of all the participants to ease access of the intervention information at anytime and anywhere without burdening the participants to carry around the hardcopy version.

### 2.5. Assessment of Glycaemic Index Nutrition Knowledge

In order to measure participants’ nutrition knowledge related to glycaemic index and glycaemic load, the previously developed questionnaire by Yusof and colleagues [10] which was further validated by Shyam [9] was adapted in this study. A pre-test was conducted to determine the reliability of these questions for use in this study. A reliability analysis using Cronbach’s alpha value of 0.70 is considered acceptable and the questionnaire is reliable for use and considered good when above 0.8 [17]. If the value is below 0.70, it indicates that the item is not reliable. Upon removal of one question, the overall Cronbach’s alpha value for this set of questionnaires was 0.76. In total, there were five questions on concepts and definition of GI and GL, four questions on the impact of low and high GI foods on post-prandial glucose and five questions on the blood glucose control and carbohydrate.

This questionnaire was employed at the beginning of the pre-intervention phase (as baseline) as well as after the completion of the intervention to make comparison on the participants’ knowledge on GI and GL diet prior to the nutrition education delivered during the intervention and observe if there any improvements after the intervention.

### 2.6. Physical Activities

Participants were advised not to increase their physical activities in this study to prevent variation of physical activity levels of participants throughout the study period. Their physical activity level was used as a control when conducting statistical analysis. The physical activity of the respondents was measured using the validated English and Malay versions of the International Physical Activity Questionnaire (IPAQ)—Short Version [18]. Participants were categorised into low, moderate and high PAL based on the IPAQ Short scoring protocol according to the participants’ metabolic equivalents per minute (MET-min) per week scores. Individuals scoring below 600 MET-min were classified as having low PAL, while those scoring above 3000 MET-min were considered as having high PAL.

### 2.7. Food Record

The participants were instructed to record the amount of food and beverages eaten each day using a 3-day food diary (two weekdays and one weekend) [19,20]. They were also required to take photos of every food item consumed within those three days and to send the photos taken to the nutritionist in charge via Telegram. Food recording was carried out during the pre-intervention period (day 2–day 4) and in the 8th week (day 58–day 60) of the intervention duration. Participants were trained in keeping food dairies during recruitment. Detailed food descriptions, including food brand names, food preparation and cooking method, were recorded. Photos were to be taken by placing money notes next to the food items to allow better estimation of size or portion of foods by the nutritionist. The research nutritionist went through all the food records with the participants to ensure completeness of the entry and to clarify any ambiguous information [12,14,21]. The food record data were analysed using DietPLUS Version3, an Excel-based Malaysian food composition database equipped with macro- and micronutrient and GI and GL calculators, which was developed by Ng and colleagues [22] and further improved by the addition of the GI and GL component by Shyam and associates [23].

### 2.8. Anthropometric and Body Composition Measurement

Parameters including height (m), weight (kg), body mass index (kg/m^2^), body fat (kg and %), muscle mass (kg) and visceral fat rating of the participants were measured. Participants were required to wear minimal clothing and all measurements were done in triplicates. Standing height without shoes was measured by using Seca 213 Stadiometer (Seca, Hamburg, Germany) to the nearest 0.1 cm. Body weight, body mass index, body fat and visceral fat rating were measured using Tanita DC-360 Body Composition Analyser (Tanita, Tokyo, Japan). The body composition analyser was set up on a flat, hard surface. The body mass index (BMI) was computed as follows: weight (kg)/height (m^2^). Standing height and body composition measurements were taken in duplicate.

### 2.9. Biochemical Analysis

Blood samples of the participants were taken for the following analysis of metabolic parameters: fasting plasma glucose (mmol/L), HbA1c (%), insulin (pmol/L), total cholesterol (mmol/L), high-density lipoprotein (HDL) (mmol/L), low-density lipoprotein (LDL) (mmol/L), triglyceride (mmol/L) and total/HDL cholesterol ratio. Insulin resistance will be assessed using homeostasis model assessment (HOMA-IR), where fasting glucose and insulin values will be inserted in the HOMA2 calculator version 2.2.3 (http://www.dtu.ox.ac.uk/homacalculator/, accessed on 18 October 2021) [24]. In total, 10 mL of blood was collected from each subject for analysis of the above mentioned metabolic parameters. Blood specimen collection was conducted by a trained phlebotomist from Pathlab and Clinical Laboratory (M) Sdn Bhd.

### 2.10. Continuous Glucose Measurement

**Figure 3:**
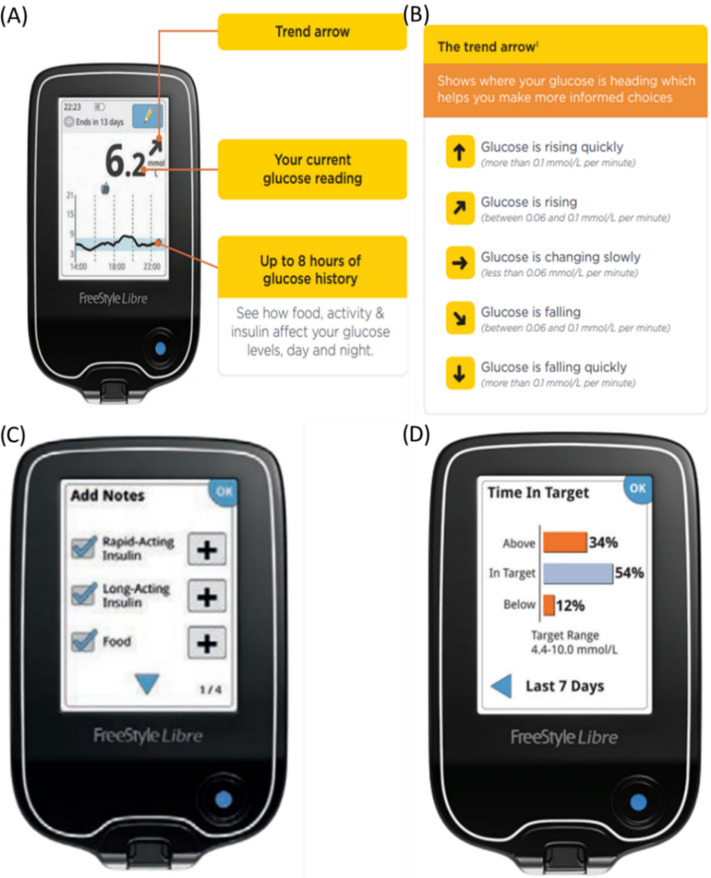
(**A**) General display of rtCGM indicating the predicted direction of glucose level as shown by the trend arrow, the current glucose reading and eight hours glucose history graph; (**B**) Description of the trend arrows; (**C**) Display option of adding notes at a specific time; (**D**) History of glucose levels based on time in targeted ranges.

The rtCGM system used in this study was Abbott Diabetes Care’s FreeStyle Libre, which is based in Alameda, CA, USA. FreeStyle Libre uses an rtCGM sensor to offer real-time continuous glucose measurements every 15 min, 24 h a day, for 14 days. The sensor has glucose oxidase impregnated in the sensor electrode, which uses a chemical reaction to convert the interstitial glucose to a signal. Information about glucose levels is then wirelessly communicated in real time to an rtCGM transmitter, where it may be monitored and downloaded for analysis. FreeStyle Libre sensor does not need to be calibrated using finger pricking as it has been readily calibrated during the manufacturing process. In this study, a nutritionist guided the participants on how to insert the sensor on their own to avoid any physical contact with the participants. They were then briefed on how to use the reader including settings of targeted glucose levels, reading of glucose levels graph, meals consumption time, history of glucose levels, predicted direction of glucose level and reminders settings, as shown in Figure 3. They were also provided with a take-home manual to refer to whenever needed. The benefits and advantages of the rtCGM system was also explained to the participants to further motivate them to utilise this device. Initialising the device takes one hour and this was then considered as day 0. If no abnormalities were observed, participants continued using the CGM sensor for the specified periods throughout the two phases accordingly.

### 2.11. Statistical Analysis

Quantitative data were analysed using Statistical Package for the Social Sciences (SPSS) software Version 23.0 (IBM, Armonk, NY, USA). Normality of data was tested using the Shapiro–Wilks test. Means and standard deviations were used to report continuous variables, while frequencies and percentages were used for categorical variables. Statistical results were considered significant at *p* < 0.05. Independent t-test was used to compare the means between the two groups, whereas paired t-test was used to compare changes within the groups before and after the intervention. The 95% confidence intervals (CIs) were calculated for the mean differences between two continuous measures. When normality and homogeneity of variances are not met, non-parametric tests were conducted. Missing data were handled using data imputation [25].

## 3. Results

### 3.1. Participants

**Figure 4:**
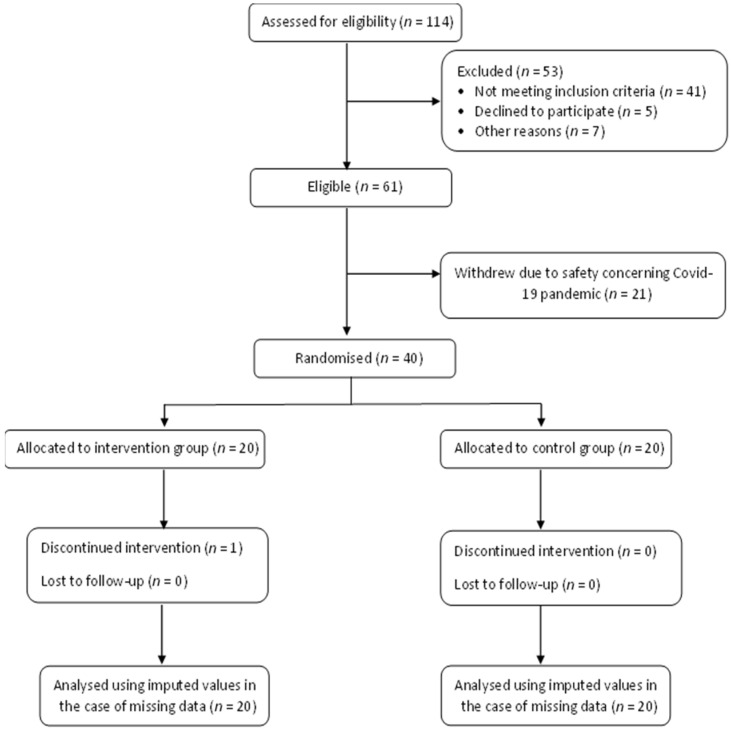
CONSORT flowchart.

Recruitment of participants for this study is summarised in Figure 4. A total of 114 individuals were assessed for eligibility. Out of the total number of individuals screened, 53 individuals were excluded due to not meeting the inclusion criteria (*n* = 41), declining to participate (*n* = 5) and for other reasons (*n* = 7), such as not having time to commit to the study plan. From the 61 eligible participants, 21 participants withdrew themselves just before the randomisation took place as they were concerned regarding their safety as the study was conducted during the COVID-19 pandemic. A total of 40 participants were then randomised equally to the intervention and control groups. During intervention, one participant from the intervention group withdrew himself due to time management.

Participants from both groups had similar (*p* > 0.05) baseline characteristics for all body composition variables and metabolic parameters as indicated in Table 1. Females were fairly well distributed between groups, as there were 12 females in the intervention group and 11 in the control group, contributing to 60% and 55% of the total participants, respectively. The participants from the groups had a non-significant difference of one kilogram in mean body weight (intervention: 77.3 ± 10.4 kg; control: 78.3 ± 14.6 kg; *p* > 0.05). Similarly, there was a one year mean age gap between the participants of the two groups with no significant difference (intervention: 26 ± 6 y/o; control: 25 ± 5 y/o; *p* > 0.05).

### 3.2. Physical Activities

Physical activities of participants from both groups were measured at baseline and post-intervention. The physical activities at baseline and post-intervention did not indicate any significant difference (*p* > 0.05) for both the intervention and control groups, as presented in Table 2. Mean physical activities in the intervention group increased by 25.9 MET-min/week (95% CI: −71.9 to 123.8; *p* = 0.59), while the control group reduced minimally by −2.6 MET-min/week (95% CI: −113.9 to 108.7; *p* = 0.96). By comparing between-group changes from baseline as shown in Table 2, there was no significant difference shown between the groups with a mean difference of −28.5 MET-min/week (95% CI: −171.9 to 114.8; *p* = 0.69).

### 3.3. Glycaemic Index (GI) and Glycaemic Load (GL) Knowledge

Participants’ GI and GL knowledge were evaluated before and after the nutrition education was delivered. Both groups indicated a significant increase (*p* < 0.001) in GI and GL scores as depicted in Table 3. Participants from the intervention group increased their mean score by 31.9% (95% CI: 21.8 to 42.0; *p* < 0.001), while the control group scored higher mean difference with 40% increment in score (95% CI: 33.2 to 56.7; *p* < 0.001). However, based on Table 3, the mean score change estimate was not significant between the two groups (mean difference: 8.08%; 95% CI: −4.92 to 21.08; *p* = 0.10).

### 3.4. Dietary Intake

Dietary intake of participants from both groups was measured at baseline and post-intervention using 3-day food diary. At baseline, the total energy intake and all macronutrients (carbohydrate, protein, fat) were similar (*p* > 0.05) between both groups. As presented in Table 4, during post-intervention, the total energy intake reduced significantly (*p* < 0.001) in both groups by about 300 kcal. Carbohydrate intake reduced significantly in terms of grams (*p* < 0.001) and as a percent of total energy (intervention, *p* < 0.001; control, *p* = 0.003) for both groups. Intake of protein in grams did not differ significantly (*p* > 0.05) for both groups. However, it indicated a significant increment (*p* < 0.001) in both groups as a percentage of total energy. Fat intake did not show any significant difference (*p* > 0.05) in grams for both groups and only a significant increment (*p* < 0.001) in the intervention group as a percentage of total energy. GI and GL both showed significant reduction (*p* < 0.001) post-intervention in both the intervention and control groups.

Comparison of changes from baseline on the dietary intake between the intervention group and the control group is shown in Table 4. The total energy intake between the groups did not indicate any significant difference (mean difference: −17 kcal; 95% CI: −53 to 17; *p* = 0.32). Both the carbohydrate intake in grams (mean difference: −15.2 g; 95% CI: −20.9 to −9.5; *p* = 0.04) and as a percentage of total energy intake (mean difference: −3.6%; 95% CI: −6.9 to −0.3; *p* = 0.03) were significantly lower in the intervention group. Protein, on the other hand, showed no significant difference both as grams (*p* = 0.64) and as a percentage of total energy intake (*p* = 0.78). Fat intake increased by 1.7 g in the intervention group and decreased by 6 g in the control group, leading to a significant difference of 7.8 g (95% CI: 1.7 to 13.9; *p* = 0.04) between the groups.

This difference is also translated in the percentage of total energy intake leading to a significant difference between the groups (mean difference: 3.7%; 95% CI: −6.9 to −0.3; *p* = 0.05). At the end of the intervention period, the glycaemic index of the intervention group reduced to 42.9 ± 4.1 (mean ± SD) and the control group to 51.3 ± 4.5. This led to a significant difference of 10 points (95% CI: −13.1 to −6.9; *p* = 0.006) change from baseline between the groups. The final glycaemic load value for the intervention group was 78.3, whereas for the control group was 95.8, indicating a significant difference from baseline of −19.9 (95% CI: −29 to 10.7; *p* = 0.008).

### 3.5. Under-Reporting of Dietary Intake

**Figure 5:**
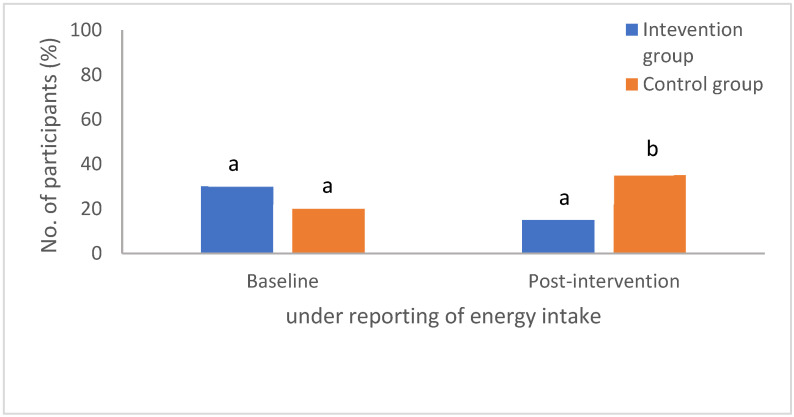
Number of participants under-reporting daily energy intake at post-intervention. Letters (a,b) indicate values are statistically significantly different (*p* ≤ 0.05) at each time point.

For the purpose of this study, under-reporting is defined as EI:BMR ratio of <1.2 as proposed by Goldberg [26]. Based on the reported 3-day food diary, a total of 6 (30%) and 4 (20%) of participants from the intervention and control groups, respectively, were found to have under-reported their dietary intake at baseline (Figure 5). Once the intervention took place, under-reporting reduced by 3 (15%) participants in the intervention group, whereas it increased by 3 (15%) participants in the control group.

The description of the post-intervention dietary intake reported by under-reporters is tabulated in Table 5. Overall, the total energy intake reported by the intervention group (1362 kcal) was higher than the control group (1143 kcal) with a mean difference of 219 kcal (95% CI: 198 to 240; *p* ≤ 0.001). The intervention group reported significantly higher carbohydrate intake (mean difference: 3.3%; 95% CI: 0.7 to 5.9; *p* = 0.009), while the control group reported significantly higher protein intake (mean difference: −2.4%; 95% CI: −4.3 to −0.5; *p* = 0.04). The fat intake did not differ (*p* > 0.05) between the groups. GI and GL were reported significantly lower in the intervention group with a mean difference of −10.7 (95% CI: −15.8 to −5.6; *p* = 0.004) and −17 (95% CI: −24.8 to −9.2; *p* = 0.003), respectively.

### 3.6. Anthropometric Measurement and Body Composition

Over a period of eight weeks, in terms of anthropometry and body composition, participants from both groups indicated significant reductions (*p* < 0.001) in body weight, BMI and fat mass as shown in Table 6. The intervention group lost an average 3.1 kg (95% CI; 2.3 to 3.8), whereas the control group lost 2.3 kg (95% CI; 1.5 to 3.0). BMI was reduced by 1.2 kg/m^2^ (95% CI: 0.9 to 1.4) in the intervention group and 0.9 kg/m^2^ (95% CI: 0.6 to 1.2) in the control group. The intervention group had a reduction of 2.8 kg of fat (95% CI: 2.1 to 3.3) while the intervention lost 2 kg (95% CI: 1.4 to 2.7). Only the intervention group indicated a significant reduction (*p* < 0.05) in muscle mass (mean: −0.3 kg; 95% CI: 0.1 to 0.6). The reduction in visceral fat rating was not significant (*p* > 0.05) in both groups.

When conducting a between-group comparison of body composition changes, body weight and fat mass showed a significant difference (*p* < 0.05) between the groups, as shown in Table 6. Weight loss among participants in the intervention group was significantly greater (*p* = 0.03) than the control group with a mean difference of −0.8 kg (95% CI: −1.4 to −0.2). Fat mass was significantly reduced in the intervention group by a mean difference of −0.7 kg (95% CI: −1.1 to −0.3) compared to the control group. Other parameters including BMI, muscle mass and visceral fat indicated no significant difference (*p* > 0.05) across time between the groups. Although no significant difference was observed, improvements were greater in the intervention group.

### 3.7. Metabolic Parameters

Metabolic parameters before and after the intervention of both groups are summarised in Table 7 below. Fasting plasma glucose in the intervention group reduced significantly over time (mean change: 0.3 mmol/L; 95% CI: 0.1 to 0.5; *p* = 0.04). However, these significant changes were not observed in the control group (mean change: 0.1 mmol/L; 95% CI: −0.1 to 0.3; *p* = 0.11). A significant reduction (*p* < 0.05) of HbA1c was reported in both groups. Insulin control parameters, fasting insulin and HOMA-IR indicated no significant difference (*p* ≥ 0.05) for both groups.

Total cholesterol reduced significantly in the intervention group (mean change: 0.3 mmol/L; 95% CI: 0.1 to 0.7; *p* = 0.03) but not the control group (mean change: 0.1 mmol/L; 95% CI: −0.7 to 1.3; *p* = 0.11). HDL improved for both groups but was only significant for the intervention group (mean change: −0.3 mmol/L; 95% CI: −0.7 to −0.1; *p* = 0.01). LDL cholesterol reduced significantly (*p* < 0.05) for both groups, whereas there were no significant changes (*p* > 0.05) for triglyceride. There was a significant reduction in the total HDL ratio for the intervention group (mean change: 0.3 mmol/L; 95% CI: 0.1 to 0.5; *p* = 0.01). However, reduction of this ratio for the control group was not significant (mean change: 0.1 mmol/L; 95% CI: −0.2 to 0.4; *p* = 0.09).

Compared to the control group, the intervention group indicated a significant (*p* < 0.05) mean change from baseline for fasting plasma glucose (mean difference: −0.1 mmol/L; 95% CI: −0.4 to −0.2; *p* = 0.04), total cholesterol (mean difference: −0.2 mmol/L; 95% CI: −0.7 to −0.3; *p* = 0.03), HDL cholesterol (mean difference: −0.2 mmol/L; 95% CI: −0.6 to −0.2; *p* = 0.01) and total HDL ratio (mean difference: −0.2 mmol/L; 95% CI: −0.3 to −0.1; *p* = 0.04) (Table 7). There were no significant differences (*p* > 0.05) between groups in mean change from baseline for HbA1c, fasting plasma insulin, HOMA-IR, LDL cholesterol and triglyceride, although improvements were greater in the intervention group.

## 4. Discussion

In this study, an RCT comparing the use of rtCGM in low GI and GL diets was conducted. Out of the 61 eligible individuals, 21 withdrew from participation prior to randomisation, as they raised their concern on the COVID-19 pandemic. At some point of time during the conduction of the RCT, the COVID-19 cases rose drastically, thus leading to a great dropout rate prior to randomisation. The RCT was conducted with strict procedures, minimising physical contact with the participants. During the intervention period, none of the participants contracted COVID-19. Baseline characteristics of participants from both groups were similar indicating no bias in randomisation.

Physical activities of both groups at baseline and post-intervention indicated no significant difference. Similarly, within-group differences were also not significant. This means that physical activity did not interfere with the intended intervention and may be excluded as a confounding factor. As stated by the WHO, the two main factors contributing to excessive body weight are physical inactivity and poor dietary intake [27]. In this study, the physical activities of participants from both groups did not change before and after the intervention. Thus, it can be assumed that any changes in body composition of the participants are not likely to be related to physical activities changes and are most likely attributed to dietary intake changes.

GI and GL knowledge of both groups were similar at baseline (intervention: 52.4%; control: 50.0%). These scores are in line with that measured by Anuar and colleagues [28] among women with gestational diabetes who scored an average of 55.6%. However, Shyam et al. (2013) reported women with gestational diabetes had a baseline score of 43%. The lower scores reported in the latter study may be influenced by the time the study was conducted, where the GI and GL concepts were newly introduced to the country where the study took place. Upon the nutrition education, scores from both groups exceeded 80%. The nutrition education provided increased their knowledge significantly surpassing the minimum score of the good category (>75%) classification applied by Anuar and colleagues [28]. The post-education scores were also similar between the groups. This may portray that there were no biases in delivering the nutrition education to the participants between the two groups.

Dietary adherence was assessed using a 3-day food diary. At the end of the intervention, both groups reduced their total daily calories intake. Within-group changes in carbohydrate intake reduced significantly in both groups, but the reduction was more significant in the intervention group, where this can be translated in terms of the GI and GL values of the diets. Although both groups had significantly reduced their daily GI and GL values of their diets, the reductions are more significant in the intervention group, in line with the reduction of the carbohydrate intake. Although the protein intake of both groups did not increase significantly, its percentage of total daily energy increased as a result of the reduction in the overall daily energy intake in the two groups. Similarly, the percentage of total daily energy from fats increased significantly in the intervention group, although the quantity of fat intake did not significantly change due to the reduction of total daily energy intake. The trend change of nutrient intake in this study is similar to that of other studies that are intended for lowering GI and GL of diets [13,20,29], where a lower percentage of carbohydrate contribution to total energy is compensated by an increase in fat and protein percentage, although intake may have not increased drastically due to the lower overall energy intake.

Post-intervention, participants from both groups had significantly reduced their body weight, BMI and fat mass. However, these reductions were more pronounced in the intervention group. In terms of metabolic parameters, fasting plasma glucose, total cholesterol, HDL and total HDL ratio were significantly improved in the intervention group only, whereas HbA1c and LDL were reduced significantly in both groups. Similarly, the reductions of HbA1c and LDL were more pronounced in the intervention group. Based on the observation above, it can be seen that the intervention group had greater improvements in the described body composition and metabolic parameters.

During the intervention, participants from both groups received similar nutrition education. It can be assumed that there was no bias in delivering the nutrition education between the groups, as both groups increased their nutrition scores significantly and the control group scored higher than the intervention group. Nutrition education can be translated into the dietary changes practiced in both groups. Significant improvements were shown in total energy intake, carbohydrate intake and daily GI and GL. However, the intervention group portrayed greater adherence to the dietary and nutrition advice given. As other main factors (physical activities and nutrition knowledge) in this study are similar between the groups, the enhanced adherence presented by the intervention group can, thus, be attributed to the use of rtCGM which was only worn by the intervention group.

As both groups reported approximately 300 kcal reduction in energy intake at post-intervention, it would have been expected that both groups would lose quite similar amounts of body weight. However, it was shown that the intervention group lost significantly higher body weight compared to the control group, which can be related to the higher rate of under-reporting of energy intake by the control group. In addition, the under-reporters from the control group further reported lower mean energy intake compared to the under-reporters from the intervention group. This implies that the mean reduction in energy intake between the groups may have not been similar. Thus, this may explain the difference in body weight loss, body composition and metabolic parameters.

As the participants from the intervention group were wearing the rtCGM sensor, it may have instilled a feeling of them being observed and monitored, thus influencing them to be more truthful in the reporting of their dietary intake, leading to the reduced number of participants under-reporting their dietary intake at post-intervention. On the other hand, the number of under-reporters in the control group increased post-intervention. This may have been caused by the belief that they were expected to practise a healthier diet by the end of the intervention. From this point of view, the rtCGM has not only motivated the participants to practise healthier diets but also improved their likelihood of truthful reporting of dietary intake. This further indicates the robustness of this tool in that it can assist in reporting dietary intake, which has been reported to greatly distort nutrition related studies which involve self-reporting of nutrients intake [30,31]. These findings are further strengthened by the participants’ feedback on the open-ended questions regarding their experience using the rtCGM. Participants stated that they had a very good experience using the device and that the rtCGM system provided them with interesting physiological information of their body which they were not aware of before. They also stated that they are more alert of their food intake, especially foods containing carbohydrates, and that the system has helped them prevent overeating. This indicates that the participants are interested and keen on knowing their glucose levels in real time. Their observation on the daily glucose levels was then translated into their behaviours. It could be observed that the daily GI and GL of the intervention group reduced significantly compared to the control group. They selected foods with lower GI and GL values to be incorporated into their daily diet. The outcome of this study further proves our earlier findings [32] that the participants’ shift in preference from high to low GI meals after the observation of their physiological changes displayed by the rtCGM can be translated into daily dietary practice by overweight and obese young adults.

## 5. Conclusions

Based on the results above, the integration of the rtCGM system into a low GI and GL diet further improved certain body composition and metabolic parameters, including body weight, BMI, fat mass, fasting plasma glucose, HbA1c, total cholesterol, HDL cholesterol and LDL cholesterol. The use of rtCGM also led to more truthful reporting of dietary intake within the intervention group, where under-reporters reduced significantly compared to the control group, which experienced an increased number of under-reporters.

The above findings indicate the robustness of this device beyond its use among diabetic patients. Overweight and obese individuals can benefit from this device and use it as a management tool for overweight and obesity and a primary prevention tool for type 2 diabetes, as it is very personalised to the individuals and provides real-time information of the individual’s physiological changes. Future studies with prolonged durations can be conducted to determine the long-term effect of this system on overweight and obese individuals.

## Figures and Tables

**Table 1:** Baseline characteristics of participants.

Variable	Intervention, (Mean ± SD) (*n* = 20)	Control, (Mean ± SD) (*n* = 20)	*p*-Value
Age, y	26 ± 6	25 ± 5	0.63
Female, No. (%)	12 (60)	11 (55)	0.72
Height, cm	164.5 ± 0.1	163.4 ± 0.1	0.85
Weight, kg	77.3 ± 10.4	78.3 ± 12.6	0.82
BMI	29.3 ± 3.7	29.9 ± 7.2	0.72
Fat mass (kg)	29.8 ± 6.9	29.7 ± 14.7	0.98
Muscle mass (kg)	44.8 ± 9.6	48.4 ± 8.8	0.76
Visceral fat rating	9.5 ± 2.6	9.3 ± 3.3	0.40
Fasting plasma glucose (mmol/L)	5.0 ± 0.5	4.8 ± 0.3	0.32
HbA1c (%)	5.2 ± 0.3	5.2 ± 0.6	0.64
Fasting plasma insulin (pmol/L)	12 ± 6.4	9.5 ± 4.4	0.16
HOMA-IR	2.5 ± 1.4	1.9 ± 0.9	0.11
Total cholesterol (mmol/L)	4.6 ± 1.2	4.5 ± 0.9	0.60
HDL cholesterol (mmol/L)	1.2 ± 0.3	1.1 ± 0.2	0.47
LDL cholesterol (mmol/L)	3.1 ± 0.7	2.9 ± 0.8	0.71
Triglycerides (mmol/L)	1.1 ± 0.7	0.9 ± 0.3	0.24
Total/HDL ratio	4.9 ± 0.9	4.7 ± 0.8	0.34

*p*-value < 0.05 indicates significance difference between the groups.

**Table 2:** Physical activities at baseline and post-intervention.

Variable	Group	Physical Activities (MET-min/Week), Mean ± SD		within-Group Differences, Mean (95% CI)	*^a^ p*-Value	between-Group Differences, Mean (95% CI)	*^b^ p*-Value
		Baseline	Post-Intervention				
Physical activities	Intervention	521.2 ± 518.3	547.2 ± 574.3	25.9 (−71.9 to 123.8)	0.59	−28.5 (−171.9 to 114.8)	0.69
	Control	498.0 ± 472.9	495.4 ± 372.1	−2.6 (−113.9 to 108.7)	0.96		

*^a^ p*-value < 0.05 indicates significance difference between baseline and post-intervention of physical activities level. *^b^ p*-value < 0.05 indicates significance difference between group change estimates of physical activities level.

**Table 3:** GI and GL knowledge scores at baseline and post-intervention.

Variable	Group	Glycaemic Index Knowledge (%) Mean ± SD		within-Group Differences, Mean (95% CI)	*^a^ p*-Value	between-Group Differences, Mean (95% CI)	*^b^ p*-Value
		Baseline	Post-Intervention				
GI and GL knowledge score (%)	Intervention	52.4 ± 21.6	84.3 ± 15.3	31.9 (21.8 to 42.0)	<0.001	8.08 (−4.92 to 21.08)	0.10
	Control	50.0 ± 26.4	90.0 ± 13.1	40.0 (33.2 to 56.7)	<0.001		

*^a^ p*-value < 0.05 indicates significance difference between baseline and post-intervention of GI and GL knowledge scores. *^b^ p*-value < 0.05 indicates significance difference between group change estimates of GI knowledge score.

**Table 4:** Dietary intake at baseline and post-intervention.

Variable	Group	Mean ± SD		within-Group Differences, Mean (95% CI)	*^a^ p*-Value	between-Group Differences, Mean (95% CI)	*^b^ p*-Value
		Baseline	Post-Intervention				
Total energy intake, Kcal	Intervention	1904 ± 284	1564 ± 237	339 (308 to 370)	<0.001	−17 (−53 to 17)	0.32
	Control	1931 ± 252	1609 ± 210	322 (302 to 341)	<0.001		
Carbohydrate, g	Intervention	277.9 ± 33.1	191.8 ± 27.3	86.1 (76.9 to 95.3)	<0.001	−15.2 (−20.9 to −9.5)	0.04
	Control	274.9 ± 40.6	204.1 ± 26.4	70.9 (53.8 to 87.9)	<0.001		
Carbohydrate, % Kcal	Intervention	58.7 ± 3.5	49.3 ± 4.0	9.5 (7.4 to 11.6)	<0.001	−3.6 (−6.9 to −0.3)	0.03
	Control	56.9 ± 4.2	51.1 ± 6.4	5.8 (2.2 to 9.4)	0.003		
Protein, g	Intervention	69.3 ± 16.3	72.6 ± 14.2	−3.3 (−8.8 to 2.2)	0.22	2.3 (7.4 to −11.9)	0.64
	Control	71.4 ± 14.0	76.9 ± 11.7	−5.6 (−13.9 to 2.9)	0.18		
Protein, % Kcal	Intervention	14.4 ± 1.6	18.6 ± 2.3	−4.2 (−5.4 to −2.9)	<0.001	−0.5 (−2.1 to 1.1)	0.78
	Control	15.0 ± 3.5	19.7 ± 2.5	−4.7 (−6.0 to −2.4)	<0.001		
Fat, g	Intervention	52.3 ± 7.8	54.0 ± 10.9	−1.7 (−4.1 to 0.6)	0.13	7.8 (1.7 to 13.9)	0.04
	Control	58.7 ± 14.5	52.7 ± 15.1	6.1 (−1.7 to 13.8)	0.11		
Fat, % Kcal	Intervention	24.9 ± 1.7	30.9 ± 2.6	−6.0 (−7.2 to −4.8)	<0.001	3.7 (1.4 to 6.0)	0.05
	Control	27.1 ± 4.7	29.4 ± 7.7	−2.2 (−6.4 to 1.9)	0.27		
Glycaemic index	Intervention	72.6 ± 4.5	42.9 ± 4.1	29.8 (27.6 to 31.9)	<0.001	−10.0 (−13.1 to −6.9)	0.006
	Control	71.0 ± 5.0	51.3 ± 4.5	19.7 (17.3 to 22.2)	<0.001		
Glycaemic load	Intervention	126.3 ± 11.8	78.3 ± 4.1	48.0 (42.5 to 53.5)	<0.001	−19.9 (−29.0 to −10.7)	0.008
	Control	123.9 ± 12.4	95.8 ± 8.7	28.1 (20.4 to 35.9)	<0.001		

*^a^ p*-value < 0.05 indicates significance difference between baseline and post-intervention of dietary intake. *^b^ p*-value < 0.05 indicates significance difference between group change estimates of dietary intake.

**Table 5:** Dietary intake of under-reporters of intervention and control groups during post-intervention.

Variable	Intervention (Mean ± SD)	Control (Mean ± SD)	between-Group Differences, Mean (95% CI)	*p*-Value
Total energy intake, kcal	1362 ± 127	1143 ± 163	219 (198 to 240)	< 0.001
Carbohydrate, % kcal	47.6 ± 3.71	44.3 ± 4.18	3.3 (0.7 to 5.9)	0.009
Protein, % kcal	19.3 ± 2.1	21.7 ± 3.4	−2.4 (−4.3 to −0.5)	0.04
Fat, % kcal	32.1 ± 3.5	32.4 ± 6.7	−0.3 (−2.7 to 2.1)	0.37
Glycaemic index	44.7 ± 4.9	55.4 ± 4.4	−10.7 (−15.8 to −5.6)	0.004
Glycaemic load	85 ± 12.9	102 ± 17.6	−17 (−24.8 to −9.2)	0.003

*p*-value < 0.05 indicates significance difference of dietary intake between intervention and control groups. Intervention, *n* = 3; control, *n* = 7.

**Table 6:** Body composition parameters at baseline and post-intervention.

Variable	Group	(Mean ± SD)		within-Group Differences, Mean (95% CI)	*^a^ p*-Value	between-Group Differences, Mean (95% CI)	*^b^ p*-Value
		Baseline	Post-Intervention				
Weight, kg	Intervention	77.3 ± 10.4	74.3 ± 11.3	3.1 (2.3 to 3.8)	<0.001	−0.8 (−1.4 to −0.2)	0.03
	Control	78.3 ± 12.6	76 ± 13.1	2.3 (1.5 to 3.0)	<0.001		
BMI, kg/m^2^	Intervention	29.3 ± 3.7	28.1 ± 3.9	1.2 (0.9 to 1.4)	<0.001	−0.2 (−0.6 to 0.2)	0.09
	Control	29.9 ± 7.2	28.9 ± 6.8	0.9 (0.6 to 1.2)	<0.001		
Fat mass, kg	Intervention	29.8 ± 6.9	27.0 ± 7.5	2.8 (2.1 to 3.3)	<0.001	−0.7 (−1.1 to −0.3)	0.04
	Control	29.7 ± 14.7	27.7 ± 14.4	2 (1.4 to 2.7)	<0.001		
Fat mass, %	Intervention	38.7 ± 7.8	36.5 ± 8.4	2.2 (1.6 to 2.7)	<0.001	−0.6 (−1.3 to 0.1)	0.07
	Control	36.8 ± 11.7	35.2 ± 12.0	1.6 (1 to 2.1)	<0.001		
Muscle mass, kg	Intervention	44.8 ± 9.6	44.5 ± 9.4	0.3 (0.1 to 0.6)	0.01	−0.2 (−0.5 to 0.2)	0.22
	Control	48.4 ± 8.8	48.3 ± 8.9	0.1 (−0.1 to 0.2	0.19		
Visceral fat rating	Intervention	9.5 ± 2.6	9.2 ± 2.8	0.3 (−0.1 to 0.6)	0.07	−0.2 (−0.5 to 0.2)	0.06
	Control	9.3 ± 3.3	9.2 ± 3.1	0.1 (−0.1 to 0.3)	0.08		

*^a^ p*-value < 0.05 indicates significance difference between baseline and post-intervention values of each body composition. *^b^ p*-value < 0.05 indicates significance difference between group change estimates of body composition.

**Table 7:** Metabolic parameters at baseline and post-intervention.

Variable	Groups	Baseline (Mean ± SD)	Post-Intervention (Mean ± SD)	within-Group Differences, Mean (95% CI)	*^a^ p*-Value	between-Group Differences, Mean (95% CI)	*^b^ p*-Value
Fasting plasma glucose, mmol/L	Intervention	5.0 ± 0.5	4.7 ± 0.3	0.3 (0.1 to 0.5)	0.04	−0.1 (−0.4 to −0.2)	0.04
	Control	4.8 ± 0.3	4.7 ± 0.4	0.1 (−0.1 to 0.3)	0.11		
HbA1c, %	Intervention	5.2 ± 0.3	5.0 ± 0.3	0.2 (0.1 to 0.5)	0.03	−0.1 (−0.1 to 0.1)	0.07
	Control	5.2 ± 0.6	5.1 ± 0.3	0.1 (0.2 to 0.4)	0.04		
Fasting plasma insulin, pmol/L	Intervention	12 ± 6.4	10.6 ± 6.5	1.4 (−1 to 2.8)	0.22	−0.3 (−3.1 to 2.7)	0.52
	Control	9.5 ± 4.4	8.4 ± 5.2	1.1 (−0.8 to 3.1)	0.24		
HOMA-IR	Intervention	2.5 ± 1.4	2.2 ± 1.4	0.3 (−0.2 to 0.9)	0.18	−0.1 (−0.7 to 0.6)	0.27
	Control	1.9 ± 0.9	1.7 ± 1	0.2 (−0.1 to 0.7)	0.17		
Total cholesterol, mmol/L	Intervention	4.9 ± 1.2	4.6 ± 1.2	0.3 (0.1 to 0.7)	0.03	−0.2 (−0.7 to −0.3)	0.04
	Control	4.7 ± 0.9	4.6 ± 0.8	0.1 (−0.7 to 1.3)	0.11		
HDL cholesterol, mmol/L	Intervention	1.2 ± 0.3	1.5 ± 0.4	−0.3 (−0.7 to −0.1)	0.01	−0.2 (−0.6 to −0.2)	0.03
	Control	1.1 ± 0.2	1.2 ± 0.3	−0.1 (−0.1 to 0.1)	0.08		
LDL cholesterol, mmol/L	Intervention	3.1 ± 0.7	2.8 ± 0.8	0.3 (0.1 to 0.5)	0.01	−0.1 (−0.1 to 0.1)	0.28
	Control	2.9 ± 0.8	2.8 ± 0.7	0.1 (0.4 to 0.6)	0.03		
Triglycerides, mmol/L	Intervention	1.1 ± 0.7	0.9 ± 0.7	0.2 (−0.1 to 3)	0.35	−0.1 (−0.1 to 0.1)	0.36
	Control	0.9 ± 0.3	0.8 ± 0.4	0.1 (−0.1 to 0.1)	0.39		
Total/HDL ratio	Intervention	4.9 ± 0.9	4.6 ± 0.8	0.3 (0.1 to 0.5)	0.01	−0.2 (−0.3 to 0.1)	0.04
	Control	4.7 ± 0.8	4.6 ± 0.8	0.1 (−0.2 to 0.4)	0.09		

*^a^ p*-value < 0.05 indicates significance difference between baseline and post-intervention values of each metabolic parameter. *^b^ p*-value < 0.05 indicates significance difference between group change estimates of metabolic parameters. Abbreviations: HDL = high-density lipoprotein; HOMA-IR = Homeostatic Model Assessment of Insulin Resistance; LDL = low-density lipoprotein.

## Data Availability

The datasets used and/or analysed during the current study are available from the corresponding author on reasonable request.
